# Cost-effectiveness analysis of clinical specialist outreach as compared to referral system in Ethiopia: an economic evaluation

**DOI:** 10.1186/1478-7547-8-13

**Published:** 2010-06-11

**Authors:** Yibeltal A Kifle, Tilahun H Nigatu

**Affiliations:** 1 College of Public Health and Medical Sciences, Jimma University, Jimma, Ethiopia; 2 Department of Monitoring, Evaluation and Research, African Medical and Research Foundation (AMREF), Addis Ababa, Ethiopia

## Abstract

****Background**:**

In countries with scarce specialized Human resource for health, patients are usually referred. The other alternative has been mobilizing specialists, clinical specialist outreach. This study examines whether clinical specialist outreach is a cost effective way of using scarce health expertise to provide specialist care as compared to provision of such services through referral system in Ethiopia.

****Methods**:**

A cross-sectional study on four purposively selected regional hospitals and three central referral hospitals was conducted from Feb 4-24, 2009. The perspective of analysis was societal covering analytic horizon and time frame from 1 April 2007 to 31 Dec 2008. Data were collected using interview of specialists, project focal persons, patients and review of records. To ensure the propriety standards of evaluation, Ethical clearance was obtained from Jimma University.

****Results**:**

It was found that 532 patients were operated at outreach hospitals in 125 specialist days. The unit cost of surgical procedures was found to be ETB 4,499.43. On the other hand, if the 125 clinical specialist days were spent to serve patients referred from zonal and regional hospitals at central referral hospitals, 438 patients could have been served. And the unit cost of surgical procedures through referral would have been ETB 6,523.27 per patient. This makes clinical specialist outreach 1.45 times more cost effective way of using scarce clinical specialists' time as compared to referral system.

****Conclusion**:**

Clinical specialist outreach is a cost effective and cost saving way of spending clinical specialists' time as compared to provision of similar services through referral system.

## Background

With the purpose of contributing to the effort of the Ministry of Health to reduce the critical shortage of specialized human resource for health, AMREF in Ethiopia has been implementing a Clinical Specialist Outreach Project (CSOP) to provide clinical specialist services in regional and zonal hospitals of the country for patients who could have been referred to central referral hospitals. The objective of the project was to provide service to patients and strengthen the capacities of ten outreach hospitals.

To achieve its objective, the project used volunteer sub-specialists and specialists with special skills from the relatively more populated areas to provide desperately needed clinical outreach services in the areas of general surgery, plastic and reconstructive surgery, orthopedic surgery, urology, ophthalmology, gynecology, pediatric surgery, neurology, radiography, gastroenterology and anesthesiology[1]. The project mobilized these volunteers from urban centers to the selected hospitals where these services were not available due to lack of skilled human power.

The project used an appointment system through which patients with cold case conditions requiring clinical specialist care will be appointed for consultation by senior physicians who will be visiting the hospitals based on their predefined schedule. During their visits, specialists manage patients and train full timer health workers working in the outreach hospitals. Specific activities that mobilized physicians performed during their visit to zonal and regional hospitals include: Screening and diagnostic services including to scheduled patients for surgery; Surgical intervention with on the jobs training for local staff and Formal lecture to build the capacity of local staff and students practicing in the outreach hospitals [2-4].

The evaluation question this study intended to answer was “Is clinical specialist outreach service a cost effective way of using scarce health expertise to provide clinical specialist care as compared to provision of such services through referral system in Ethiopia?”

## Methods

The main factor determining the outcome of interest, access to clinical specialist services, is the availability of limited number of specialists in the country. Considering this, the main effectiveness measure which is directly related with our outcome of interest, for this economic evaluation was “number of patients receiving clinical specialist services within a defined time of clinical specialists spent for this purpose”.

As the perspective is societal, the analysis considered the costs encored on different constituents participating in the provision of clinical specialist services. The costs are categorized into five exclusive categories: Direct medical cost, direct non-medical cost on patients and care takers/companions, indirect cost on patients and care takers/companions, indirect cost on voluntary clinical specialists, and Project cost to organize outreach activities.

The gain and loss by participating hospitals associated with mobilization of staff from central referral hospitals to Outreach Hospitals was ignored as we are considering societal perspective which makes the loss by the central referral hospitals to be compensated with the gain by outreach hospitals.

This study has taken two major assumptions: The technical quality of specialist care provided to patients and thus treatment outcomes are assumed to be equal for both of the alternative strategies; and if CSOP was not in place, referral to central referral hospitals would have been the only option to treat the patients.

Timeframe is the period over which intervention costs are calculated and analytic horizon refers to the period over which effects of interventions will be measured. For this particular study both the timeframe and analytic horizon are similar with the implementation period of clinical specialist outreach project which covers the period from 1 April 2007 to 31 December 2008.

### Study area and period

Included in the study were four outreach hospitals where the Clinical Specialist Outreach Project was adequately implemented and three central referral hospitals from which clinical specialists were mobilized. The period of data collection was from 4 to 24 February 2009.

### Source and Study Populations

Patients who received clinical specialist services with surgical interventions for orthopedic, plastic, urologic or gynecologic problems at outreach hospitals in the regions and central referral hospitals in Addis Ababa were the source population. The study population included two categories of sampled patients selected from the source population: Sample patients who received clinical specialist services most recently in the four outreach and three referral hospitals including those who get operated for problems related to urologic, gynecologic, and orthopedic and plastic surgeries; and Post-operative patients who received clinical specialist services from the four outreach and three referral hospitals during the three weeks period of data collection.

### Sample size and sampling technique

Purposive sampling was used to select four outreach hospitals from the ten project hospitals. The purpose was to include hospitals in which the project was adequately implemented and at the same time better represent the geographical distribution in relation to Addis Ababa. Based on these criteria four outreach hospitals were selected: Yirgalem, Adama, Nekemt, and Felege Hiwot Hospitals. For collection of data at central referral hospital level, the three hospitals from which clinical specialists were being mobilized were selected: Black Lion, St Paul and Yekatit Hospitals.

Ten charts for each surgical intervention undertaken through clinical specialist outreach project were taken for chart review. The types of procedures were those surgical procedures performed through the outreach project. Selection of charts of patients was based on date procedure performed; ten charts of patients who received service most recently were included. As the specialist outreach were for five days at a time, two charts of a day were taken.

All post operative patients who get to central referral hospitals through referral from zonal/regional hospitals and received surgical interventions during the three weeks data collection period for reasons similar with those intervened through the clinical specialist outreach project were included for patient interview.

Patients for interview were identified from surgical and gynecology wards of the selected central referral hospitals. The inclusion criteria for patient interview were: Post operative patient after surgical procedure related to the four specialty areas (urologic, gynecologic, and orthopedic and plastic surgeries); and patient coming referred from a hospital outside of Addis Ababa.

### Data Collection

The data collection team included the principal investigator, one general practitioner to review patient charts, one nurse to interview patients and one project staff to facilitate field work. All data collectors were trained by the principal investigator prior to the data collection period.

The data collection tools include: Clinical Specialist outreach visit details sheet, Clinical specialist activities summarizing sheet, Patient interview questionnaire, Patient Record reviewing tool, Interview guide for specialists, Project cost estimation tool, and Interview guide for outreach hospital focal persons. Two major methods of data collection were used: Review of documents(patient records, reports, registration books and financial documents) and interview (Patient interview, and Expert interviews).

### Ethical consideration

The proposal has been reviewed and got ethical clearance by the Ethical Review Board of Jimma University to ensure the propriety standards of the evaluation. Informed consent was obtained from all participant patients in the data collection process prior to any attempt to collect data.

### Data analysis

Analysis of the cleaned data set was done by using SPSS 16.0. Data from different sources get linked during analysis and Microsoft Excel was used to calculate the final summary values and results were presented in tables, graphs and narrative descriptions. Sensitivity analysis was done to assess how the result of the analysis could change based on the values of some selected independent variables with a potential to change over time and across different contexts.

## Results

### Performance of alternative strategies

Clinical specialists mobilized for surgical interventions have been spending 90% of their time doing plastic, gynecology, orthopedic and urologic surgery and the rest 10% of their time while doing other activities including lecture for students and conducting non-surgical patient management.

The results of this study showed that a total of 23 clinical specialist outreach visits were made in 21 visits to the four hospitals. During these visits a total of 139 specialist days (calculated as sum of number of days spent by each specialist in outreach hospitals) were spent, and 101 (72.7%) of the spent specialist days were for service provision in outreach hospitals while the rest 38 (27.3%) were spent for traveling. Considering this it can be estimated that 14 (10%) of the total 139 clinical specialist days spent was for activities other than surgery. A total of 125 clinical specialist days were spent to conduct surgery in the four sample outreach hospitals

During the 21 specialist visits made to the four outreach hospitals, a total of 432 surgeries were performed by mobilized specialists. It was found that 100 surgeries were performed by trained specialists. A total of 532 patients have been operated for diseases which would have required referral to Central Referral Hospitals had it not been for the outreach project. And this makes the effectiveness of Clinical Specialist Outreach to be 4.26 surgeries per a day of a clinical specialist spent when the on the jobs training role of the outreach project is considered and 3.46 when the on the jobs training role is not considered.

The average number of surgeries conducted per a day of a clinical specialist in the central referral hospitals is three to four. This response was consistently mentioned by specialists from the four specialty areas. From this it can be estimated that 438 patients could have been served during the 125 specialist days invested for clinical specialist outreach project to the four hospitals.

### Cost of alternative strategies

#### Medical Cost of Surgical Procedures

Medical costs include costs of pre-oprative care, costs of the surgical procedure and costs of post-oprative care including cost of drugs and diagnostic materials. The weighted average medical cost of surgical procedures conducted through CSOP was Ethiopian Birr (ETB) 1,124.93 per patient which was ETB940.16, ETB1315.87, ETB1074.57 and ETB 1470.36 for Gynecologic Surgery, Orthopedic Surgery, Plastic Surgery and Urologic Surgery, respectively.

#### Direct non-medical cost on patients and care takers

This cost category includes costs of travel and accomodation of the patient and caretakers. To determine the average direct non-medical and indirect costs encored on patients and care takers, 38 post-operative patients who get operated through referral system for disease conditions similar to those served through outreach project were interviewed. The interviewee included 21 (55.3%) females and 18 (44.7%) males (Table 1).

**Table 1:** Socio demographic characteristics of patients interviewed

**Variable**	**Response**	**Number**	**Percent**
Age	Below 18 years old	11	28.9%
	18 years or older	27	71.1%
	**Total**	**38**	**100.0%**
Sex	Female	21	55.3%
	Male	17	44.7%
	**Total**	**38**	**100.0%**
Educational status	Can't read and write	19	70.4%
	Can read and write	7	25.9%
	Attended formal education	1	3.7%
	**Total**	**27**	**100.0%**
Occupation	Farmer	6	19.4%
	Trader/Merchant	3	9.7%
	Government employee	4	12.9%
	House wife	5	16.1%
	Student	4	12.9%
	House maid	2	6.5%
	Daily laborer	3	9.7%
	Hand craft	1	3.2%
	Other	3	9.7%
	**Total**	**31**	**100.0%**

The direct non-medical cost of surgical interventions was 1,633.00 when patients receive services through clinical specialist outreach as compared to 3,358.34 when similar services are provided at central referral hospitals through referral system. This shows more than 50% reduction of direct non-medical cost when patients receive clinical specialist services at outreach hospitals as compared to that at central referral hospitals.

#### Indirect Cost on Patients and Care Takers

This is the cost of days lost for patient and the care takers. Average monthly income of patients and care takers above the age of 18 years was found to be 593.3ETB, making an average daily income of 19.8ETB. On average, patients spend 0.66 days to travel from their home to referring/outreach hospital and 1.87 days to travel from their home to central referal hospital. The average duration of stay at hospitals during different stages of care were: 9.6 days at referring/outreach hospital to get diagnostic services and referral, 4.85 days to see a doctor and get appointment for surgery at central referal hospitals and 13.85 days and 12.04 days at central referal hospital before and after operation is conducted.

In the clinical specialist outreach approach, patients spend 0.66 days to travel from home to the outreach hospital, 9.6 days to get diagnostic services and get appointment for outreach services, 0.66 days to travel from outreach hospital back to home, 0.66 days to travel from home to outreach hospital on date of clinical specialist outreach service, 3 days for waiting time after admission and preoperative care, 12.04 days for operative and Post-operative care and 0.66 days to travel from outreach hospital back to home.

About 71% of the patients and 100% of the patient companions were above the age of 18 years old. Considering the average monthly income of economically active patients and care takers, which is 19.80ETB, the average loss of productivity for patients and care takers was found to be ETB 2,040.85 and ETB 1,336.94 per a patient receiving care through referral and clinical specialist outreach project, respectively.

#### Project cost of clinical specialist outreach

The project cost in this study is the cost incurred in the coordination of specialist visits. Review of project fianancial documents showed that the total expenditure of the project during its life was ETB 2,153,773.15. And ETB 353,215.21 (16.4%) of the project's expenditure was made for activities during the preparatory phase and the rest ETB 1,800,557.94 (83.6%) was spent during the actual implementation period. The total project cost for surgical interventions is estimated to be ETB506,067.80. The total number of surgeries conducted was 1,629 and this makes the average project cost per surgery ETB 310.66.

#### Loss of income and expenses by mobilized clinical specialists

Specialists were loosing income from their extra hour private businesses. Specialists loose an average daily income of ETB 750 with a possibility to range between ETB 500 and ETB 1000 when they participate with permission from their base hospital. More over, specialists estimated their daily extra expenditure because of their movement at an average rate of ETB 300 per day. The project was providing reimbursement of ETB 650 per day. These cost estimates make an average daily loss of income of ETB 400. The average number of surgeries made per a day of a specialist was 4.26. This makes an investment of volunteer specialists per a patient operated to be ETB93.90 9 (Table 2). The major cost categories that contributed to the difference in the two alternatives are direct non-medical costs for patients and care takers and indirect costs for patients and caretakers which were highest for the referral approach. These costs get higher because of the larger distances.

**Table 2:** Costs and outcomes of alternative strategies for 125 specialist days invested

**Cost Category**	**Clinical Specialist Outreach**			**Referral System**		
	**Unit cost in ETB**	**No of operations**	**Total cost in ETB**	**Unit cost in ETB**	**No of operations**	**Total cost in ETB**
Direct medical cost	1,124.93	532.00	598,462.76	1,124.93	438.00	492,719.34
Direct non-medical cost	1,633.00	532.00	868,756.00	3,358.34	438.00	1,470,952.92
Indirect cost on patients and care takers	1,336.94	532.00	711,252.08	2,040.00	438.00	893,520.00
Indirect cost on specialists	93.90	532.00	49,954.80	0	438.00	0.00
Project cost	310.66	532.00	165,271.12	0	438.00	0.00
Total cost per patient operated	4,499.43	532.00	2,393,696.76	6,523.27	438.00	2,857,192.26

Provision of clinical specialist services through outreach was found to be more effective and less costly. For 125 clinical specialist days invested clinical specialist outreach enables provision of specialist services for 532 patients which is 121.5% of that expected if the same specialist days were spent in the operation rooms at the specialists' base hospitals. Moreover, the cost of providing clinical specialist service for one patient was found to be 4,499.43 for clinical specialist outreach services as compared to 6,523.27 for referral services showing 31.0% reduction of cost (Table 3).

**Table 3:** Summary of unit costs of clinical specialist services for alternative strategies

**Cost Category**	**CSOP**		**Referral System**	
	**Amount in ETB**	**%**	**Amount in ETB**	**%**
Direct medical cost	1,124.93	25.00%	1,124.93	17.24%
Direct non-medical cost	1,633.00	36.29%	3,358.34	51.48%
Indirect cost on patients and care takers	1,336.94	29.71%	2,040.00	31.27%
Indirect cost on specialists	93.90	2.09%	0.00	0.00%
Project cost	310.66	6.90%	0.00	0.00%
**Total**	**4,499.43**	**100.00%**	**6,523.27**	**100.00%**

### Cost-effectiveness of the alternatives

This makes an average cost effectiveness ratio of 1.45 showing that clinical specialist outreach service is 1.45 times more cost effective way of using scarce clinical specialists to provide surgical specialist services for patients outside of Addis Ababa as compared to provision of similar services through referral linkage between hospials. Further analysis of the different cost components showed that voluntary participation of clinical specialists costing ETB 1.0 with an investment of ETB 3.3 to coordinate activities will save ETB 25.9 for pateints while receiving clinical specialist services. Besides, 93.5% of patients reported that they will prefer to be served by nearby hospitals at a cost which is equivalent to the amount they paid to get the services through referral.

### Sensitivity analysis

Exclussion of results due to the on the jobs training role of Clinical Specialist Outreatch, consideration of the maximum value of estimated performance of central referal hospitals and variation in direct medical cost of procedures, analyzed separately, didn't change the conclussion that clinical specialsit outreach is more cost effective than referral system in using the time of scarce clinical specialists. Changes in project cost and loss of income by voluntary specialists were also found not to change this conclussion untill the increment gets as high as five times of the current estimates, provided that other things keep constant.

## Discussion

In this study, we found that clinical specialist outreach is both cost effective and cost saving, from societal perspective, approach to provide specialist surgical services to pateints outside of Addis Ababa who otherwise could have been referred to central referral hospitals. Additional investment from preoviers side including voluntary participation of clinical specialists costing ETB 1.0 and program cost of ETB 3.3 to coordinate activities was found to save ETB 25.9 for pateints and care takers. The difference between the additional cost required from the providers side and the amount saved for patients indicates the possibility to introduce user fee as a mechanism to ensure sustainability.

Similar studies from Ethiopia were not available for comparison. The advantages to pateints and care takers observed in this study are found consistent with those reported by other studies elsewhere. A systematic review of outreach clinics in primary health care in the UK revealed that outreach services have the potential to improve access to health care with no compromize in patient outcomes [5,6]. Ease to access, treatment near home and shorter waiting time were the major advantages reported in different studies[7,8]. In agreement with these studies, we found that outreach service was able to reduce the direct non-medical cost and indirect cost of care on pateints and their attendants by a factor of half and two third, respectively. These categories of costs were reported as major barriers of timely care[9], indicating the potential of outreach services to improve access to specialist health care in Ethiopia.

This study provides a basis to expand and institutionalize clinical specialist outreach services in Ethiopia with a condition that there will be no change in the quality of care and treatment outcomes. In situations where this assumption is in question, further studies are required.

## Conclusion and recomendations

Clinical specialist outreach is found to be a cost effective and cost saving approach of using scarce clinical specialists for provision of clinical specialist services to people outside of Addis Ababa as compared to provision of similar services through referral system. The time of scarce clinical specialists basing in central referal hospitals can be used to provide clinical specialist services to an average of 4.26 pateints per specialist-day at a cost of ETB 4,499.43 per patient through clinical specialist outreach or 3.5 pateints per specialist day at a cost of ETB 6,523.27 through referral system.

Clinical specialist outreach is a more effective and less costly way of providing clinical specialist services to patients with disease conditions that require referral to central referal hospitals as compared to provision of such services through referral system. Voluntary participation of a clinical specialist costing ETB 1.0 and an investment of ETB 3.3 to coordinate voluntary services was found to save ETB 25.9 for pateints and care takers. Thus clinical specialist outreach should be considered as one of the potential strategies to improve access to care and treatment services for the people of Ethiopia living outside of the capital where such specialist services are not available.

To ensure sustainability of services and further improve the cost effectiveness of the strategy, voluntary clinical specialist outreach services should be institutionalized in the current health service delivery system of the country.

## Competing interests

The authors decalre that they have no competing interests.

## Authors' contributions

Both authors have involved in the protocol development, tool development, data collection, data analysis, report writting and manuscript preparation as well. Both authors have read and approved the final manuscript.
